# Cell cycle dynamics of mouse embryonic stem cells in the ground state and during transition to formative pluripotency

**DOI:** 10.1038/s41598-019-44537-0

**Published:** 2019-05-29

**Authors:** Ariel Waisman, Federico Sevlever, Martín Elías Costa, María Soledad Cosentino, Santiago G. Miriuka, Alejandra C. Ventura, Alejandra S. Guberman

**Affiliations:** 1 Universidad de Buenos Aires, Facultad de Ciencias Exactas y Naturales, Departamento de Química Biológica, Instituto de Química Biológica (IQUIBICEN), Laboratorio de Regulación Génica en Células Madre, Buenos Aires, Argentina; 2 CONICET - Fundación para la Lucha contra las Enfermedades Neurológicas de la Infancia (FLENI), Laboratorio de Investigación de Aplicación a Neurociencias (LIAN), Buenos Aires, Argentina; 3 CONICET - Universidad de Buenos Aires, Instituto de Fisiología, Biología Molecular y Neurociencias (IFIBYNE), Buenos Aires, Argentina; 4 Universidad de Buenos Aires, Buenos Aires, Argentina; 5 Universidad de Buenos Aires, Facultad de Ciencias Exactas y Naturales, Departamento de Fisiología y Biología Molecular y Celular, Buenos Aires, Buenos Aires, Argentina

**Keywords:** Cellular imaging, Data mining, Cell growth, Embryonic stem cells, Self-renewal

## Abstract

Mouse embryonic stem cells (mESCs) can be maintained as homogeneous populations in the ground state of pluripotency. Release from this state in minimal conditions allows to obtain cells that resemble those of the early post-implantation epiblast, providing an important developmental model to study cell identity transitions. However, the cell cycle dynamics of mESCs in the ground state and during its dissolution have not been extensively studied. By performing live imaging experiments of mESCs bearing cell cycle reporters, we show here that cells in the pluripotent ground state display a cell cycle structure comparable to the reported for mESCs in serum-based media. Upon release from self-renewal, the cell cycle is rapidly accelerated by a reduction in the length of the G1 phase and of the S/G2/M phases, causing an increased proliferation rate. Analysis of cell lineages indicates that cell cycle variables of sister cells are highly correlated, suggesting the existence of inherited cell cycle regulators from the parental cell. Together with a major morphological reconfiguration upon differentiation, our findings support a correlation between this *in vitro* model and early embryonic events.

## Introduction

Mouse embryonic stem cells (mESCs) are cells derived from the inner cell mass of the developing blastocyst^1^. They are able to self-renew indefinitely *in vitro* while preserving the developmental potential to reconstitute all embryonic lineages, ability that has been termed ‘naive’ pluripotency^2^. Among the multiple parameters that regulate cell differentiation and lineage commitment, the cell cycle has emerged as a major determinant for cell fate decisions. In this sense, a key observation was that both mouse and human ESCs are primed to initiate cell fate decisions when differentiating signals are received in the G1 phase of the cell cycle^3–5^. mESCs cultured in serum-based media display an unusual cell cycle structure characterized by fast doubling times and a truncated G1 phase that lasts approximately 2 h. As lineage commitment begins, the cell cycle length increases, mainly due to an elongation of the G1 phase. Interestingly, there have been many reports linking the cell cycle structure of mESCs with the maintenance of pluripotent state, although there is still ongoing debate in the field^6^.

Historically, mESCs have been routinely cultured in media containing fetal bovine serum (FBS) and the cytokine LIF (FBS/LIF medium), which provides the environmental cues to maintain pluripotency *in vitro*. However, cells cultured in the presence of FBS display high population heterogeneity at the transcriptional, epigenetic and developmental levels^7^. In recent years, the discovery of two small molecules (‘2i’) that can directly inhibit the differentiation pathways of MEK1/2 and GSK3α/β has allowed the establishment of defined culture conditions in which mESCs present highly homogeneous populations^8^. mESCs grown in the defined media N2B27 in the presence of 2i and LIF (2i + LIF medium) have been shown to be transcriptionally similar to the pre-implantation epiblast cells of 4.5 days and are thus referred to be in a naive ground state of pluripotency^9,10^. Although this culture conditions represent the current ‘gold standard’ to maintain mESCs *in vitro*, the cell cycle of cells in the pluripotent ground state has not been studied in detail^6^.

When the signals that maintain the naive pluripotency are removed, mESCs embark in differentiation processes that recapitulate early embryonic development and that can give rise to terminally differentiated cells^11^. Interestingly, the different categories of pluripotent cells have recently broadened with the description of pluripotent cell types that resemble different stages of the post-implantation development, prior to lineage commitment. Epiblast stem cells (EpiSCs), for instance, are similar to pre-gastrulating epiblast cells and can be differentiated from mESCs or directly derived from post-implantation blastocysts^12^. These cells are also pluripotent, but are said to be in a ‘primed’ state of pluripotency, reflecting the incipient expression of lineage-specification factors^13^. Although the cell cycle of EpiSCs has not been extensively studied, it has been reported that transition to primed pluripotency is also accompanied by a lengthening of the G1 phase^3^. Recently, other types of pluripotent cells have been described, which are directly differentiated from naive mESCs and resemble early post-implantation epiblast cells of 5.5–6.0 days. They are obtained within 24–48 hours upon withdrawal of self-renewal stimuli alone or in combination with inductive Activin and bFGF signaling^14,15^. This transition is characterized by the downregulation of the naive pluripotency markers Nanog, Klf4 and Esrrb and by the upregulation of post-implantation pluripotency markers FGF5, Oct-6 and Dnmt3A. The term ‘formative’ pluripotency has recently been coined to distinguish the pluripotency status of these cells from more developmentally advanced primed cells such as EpiSCs and their embryonic counterpart^16^. Although transition from the ground state to the formative state represents a powerful developmental model to investigate genetic and epigenetic mechanisms that regulate pluripotency and differentiation, a detailed analysis of the cell cycle structure and dynamics during this transition has not yet been performed.

In this work, we aimed to present a detailed characterization of the cell cycle dynamics of mESCs in the naive ground state and during the transition to formative pluripotency at single cell resolution. By performing prolonged live imaging experiments of mESCs bearing cell cycle reporters, we show that mESCs in the ground state of pluripotency display a cell cycle structure comparable to the reported for mESCs in FBS/LIF. Importantly, we demonstrate that transition to formative pluripotency is accompanied by a shortening of the cell cycle at very early stages. We also show that cell cycle parameters are correlated between cells of the same lineage, being greater for sister cells, both in ground state conditions and during its dissolution. Finally, we characterize the morphological dynamics of cell colonies during differentiation, which correlates with the structural reconfiguration of cells that takes place during embryonic implantation.

## Results

**Figure 1:**
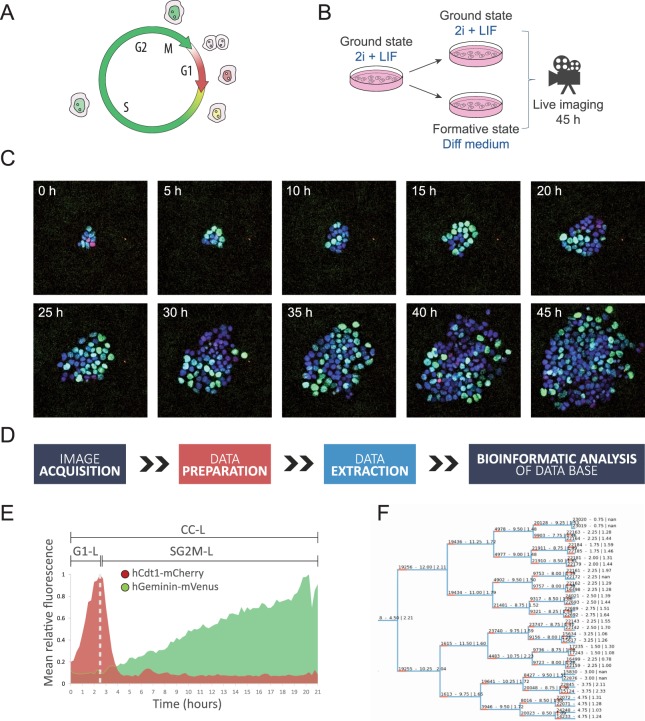
Live imaging and bioinformatic data acquisition. (**A**) Diagram of the Fucci System. (**B**) Experimental approach. (**C**) Time-lapse images of a representative FUCCI-H2B colony in naive ground state conditions growing for 45 h. Fluorescent images are merged compositions of the three channels corresponding to hCd1-mCherry (G1), hGeminin-mVenus (SG2M) and H2B-mCerulean (nuclear marker). (**D**) Schematic of the different steps performed to acquire and analyze single cell live imaging data. (**E**) hCd1-mCherry and hGeminin-mVenus fluorescence dynamics for a representative cell over time. CC-L was defined as the length of time since a cell was born and up to its division, deduced by mitotic events analyzed with the H2B nuclear marker. G1-L was defined as the time since a cell was born and up to the local maximum of the hCd1-mCherry fluorescence, while SG2M-L was calculated as the difference between CC-L and G1-L. (**F**) Representative dendrogram reconstructed for a lineage of cells growing in differentiating conditions.

To precisely characterize the cell cycle dynamics of mESCs in the ground state of pluripotency and as they transition to the formative state, we used a cell line previously engineered to express the FUCCI cell cycle reporters together with an H2B nuclear marker. The FUCCI system allows to faithfully identify cells in the G1 and in the S/G2/M phases of the cell cycle by differentially expressing two fluorescent markers fused to cell cycle regulated proteins (Fig. 1A)^17^. We have previously validated that this engineered cell line faithfully reports the cell cycle phases and efficiently differentiates from the ground state to formative pluripotency^18^.

To obtain a comprehensive picture of the cell cycle dynamics of mESCs, we performed live imaging experiments on several colonies over the course of 45 hours, as cells were maintained in the ground state and as they transitioned to formative pluripotency (Fig. 1B). To this end, cells were clonally seeded into culture plates, allowed to form small colonies over the course of 48 h and then subjected to live imaging for the following 45 h either in 2i + LIF medium or in differentiation medium (‘Diff medium’, Fig. 1C, Video S1). We applied a semi-automatic bioinformatic strategy to track cells and their lineage connections over time (Figs 1D, S1, see Supplementary Materials and Methods). This allowed us to automatically extract cell cycle parameters from individual cells based on the FUCCI and H2B reporters, such as the length of the complete cell cycle (CC-L), of the G1 phase (G1-L) and the combined length of the S/G2/M phases (SG2M-L) (Fig. 1E). Furthermore, it allowed the reconstruction of the cell lineages within colonies, both in the naive ground state and during differentiation to formative pluripotency (Fig. 1F). After manually curating all the events, we generated a data base with detailed information of more than 20 colonies, 120 cell lineages and 3000 cells for both culture conditions, which was used as a starting point to analyze the cell cycle dynamics. Of note, only 42/1387 (3%) and 17/1601 (1%) of the cells entered apoptosis in 2i + LIF and during the transition to formative pluripotency, respectively, indicating low phototoxicity in our experimental setup.

### Transition from the naive ground state to formative pluripotency is associated to a shortening of cell cycle phases

Multiple reports describe the cell cycle properties of mESCs cultured in FBS/LIF medium^19,20^. However, up to date there are no comprehensive studies analyzing the cell cycle dynamics of mESCs cultured in the naive ground state or during the transition to formative pluripotency. Thus, we first decided to analyze the cell cycle distribution of cells maintained in ground state conditions or induced to exit this state. To accurately quantify the cell cycle parameters, we initially limited our analysis to cells with a complete cell cycle in our database, i.e., for which we had a record of the time of birth and time of division. This excluded cells that were already present at the start of the video (generation 1) and cells at the end of the video, for which it was not possible to establish the CC-L.

**Figure 2:**
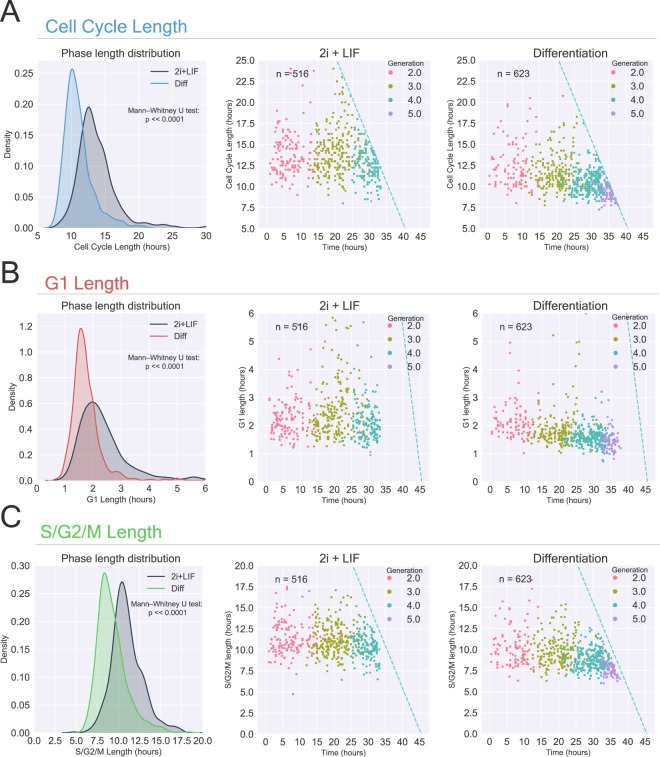
Transition to formative pluripotency is coupled to a reduction in CC-L, G1-L and SG2M-L. (**A**) *Left panel*, density distribution of single cell CC-L comparing cells maintained in the naive ground state and during the transition to formative pluripotency. Statistical differences between groups were assessed by a Mann Whitney U test. *Center* and *right panels*, plots showing time of birth vs. CC-L for 2i + LIF and Diff treated cells, respectively. Generation numbers for the different cells is shown in colors. Dashed lines show the limit of observable events. (**B,C**) Same as in (**A**) but for G1-L and SG2M-L. N = 516 and 623 cells for naive ground state and differentiation, respectively.

Mammalian embryonic stem cell differentiation is generally associated to a lengthening of the cell cycle, mostly due to an increase in the duration of the G1 phase^3,21^. For this reason, we were surprised to find that while mESCs in 2i + LIF displayed a median cycle length of 13.25 h, cells induced to differentiate displayed a median of 10.5 h, difference that was statistically significant according to a Mann Whitney U test (p ≪ 0.0001) (Fig. 2A, left panel). As this observation suggests, this was also accompanied by an increase in the number of cells over time in the differentiation condition (Fig. S2A). To further analyze this behavior, we plotted the cycle length versus time of birth of each cell, while also visualizing the cell generation number (Fig. 2A, center and right panels). This analysis showed that while cells in 2i + LIF presented a stable CC-L over time, cells induced to differentiate rapidly decreased their cycle duration, with this behavior being further exacerbated over time. This also resulted in a significant number of cells that reached the 5th generation by 45 h in the Diff condition (12% of total data), while this percentage was insignificant for cells maintained in the ground state (0.4% of the total). Interestingly, a statistically significant shortening of the CC-L was already evident for differentiating cells in the second generation (p < 0.01) (Fig. S2B).

Since the length of the videos is finite, there is a limit to the CC-L a cell can have and still be completely tracked, and this is continuously reduced as time progresses (see dashed lines in Fig. 2A). In other words, if a cell is born at 20 h of imaging and its CC-L was larger than 25 h, given the imaging length of 45 h, the cell tracking would be incomplete and would thus be excluded from our analysis. Thus, as we reach the end of the time lapse experiments there is a bias to only study cells with a short CC-L, which would underestimate the mean value of the cell cycle variables for cells in later generations. To overcome this bias, we decided to calculate the survival function for the CC-L by applying the Kaplan Meier estimator to cells in the different generations^22^. The survival function is the probability of a cell to ‘survive’ over time - here, to complete the cycle - and it is estimated by dividing the number of cell divisions at each time over the number of remaining cells. This estimator takes information from both the complete and incomplete cells, allowing to estimate the median value of a specific variable for each generation. By applying this method we found that, while in 2i + LIF the median CC-L was approximately 13.5 h for all generations analyzed, for differentiating cells we obtained a statistically significant decreasing median CC-L value of 12, 11.25, 10.5 and 9.5 h for the 2nd, 3rd, 4th and 5th generation, respectively (Fig. S2C,D).

We next wondered if the difference in CC-L observed between the two culture conditions was solely caused by a modification in the length of the G1 phase. To this end, we analyzed the G1-L and the SG2M-L distributions in the two conditions. Interestingly, both G1-L and SG2M-L distributions were shortened upon the induction of differentiation (Fig. 2B,C, left panel). Similar to the behavior of the CC-L in ground state conditions, the median G1-L and SG2M-L were stable overtime when cells were cultured in 2i + LIF but were continually reduced when cells were induced to differentiate (Fig. S2D). Furthermore, by analyzing the median phase length, we observed that regardless of the culture condition and the generation number, the ratio of G1-L to SG2M-L was constant, i.e., changed in the same proportion for both variables (Fig. S2E). This indicates that, at least in this experimental system, the length of the cycle phases is highly correlated. Importantly, the greatest absolute contribution to the overall change in CC-L resulted from the shortening in the SG2M-L, which is substantially longer than G1-L. This challenges the view that the modification of the total cell cycle length is mainly a consequence of changes in length of the G1 phase.

Overall, our time lapse analysis shows that as mESCs transit from naive to formative pluripotency, there is a reconfiguration of the cell cycle that promotes an increased proliferation rate, which is readily detected during the first 10 h since differentiation stimulus (cells in the 2nd generation). To further validate these results, we analyzed the incorporation of the nucleoside analog EdU^23^ followed by propidium iodide staining at 0, 24 and 48 h during the transition to formative pluripotency. This analysis showed a statistically significant increase in the proportion of cells in the S-phase at 48 h (Fig. S3A), thus corroborating our previous findings. Interestingly, we did not observe a statistically significant increase in the proportion of S-phase cells at 24 h, suggesting that this technique is not sensitive enough to detect changes that were already evident by single cell time lapse analysis. Similar results were obtained when analyzing two other independent mESCs lines, 46 C and Ainv15 cells (Fig. S3B,C), suggesting that the increased proliferation rate during the transition from naive to formative pluripotency is not cell line specific, but rather a general behavior.

### The length of cell cycle phases is highly correlated between sister cells

The cell cycle length has been shown to be highly correlated between cells of the same lineage, including mESCs cultured in FBS/LIF^24,25^. This could be a consequence of the direct inheritance of cell cycle regulators or specific epigenetic marks, but a precise mechanism has not yet been identified. We thus decided to assess the lineage correlation of cell cycle parameters in mESCs maintained in ground state conditions and during differentiation, while extending the analysis to the G1-L and SG2M-L.

**Figure 3:**
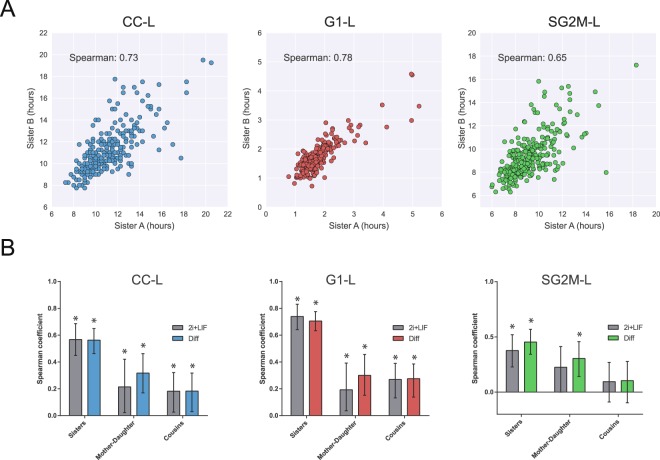
Lineage correlation of cell cycle variables. (**A**) Correlation plots for the CC-L, G1-L and SG2M-L variables for pairs of sister cells cultured in differentiating conditions. Global Spearman coefficients are shown in the plots. N = 596 cells analyzed (298 pairs of sisters). (**B**) Bootstrap calculated Spearman correlation coefficients for sister cells, mother-daughter and cousin cells maintained in ground state conditions or in the transition to formative pluripotency. Error bars represent the 95% confidence interval and * indicate p-value < 0.05 according to the bootstrap analysis.

Initial assessment of cell cycle parameters showed a high correlation between sister cells, quantified by the Spearman rank coefficient (Fig. 3A). Such high correlation, however, could be biased, especially in differentiating cells since they progressively reduce their cell cycle parameters, which could generate an overestimation of the correlations when analyzed as a whole (Fig. S4A). Another source of bias is the fact that different colonies - which are composed by cells of the same lineage - possess different mean values for the cycle variables and slightly different number of cells (data not shown). Again, this compartmentalization of the data could bias the correlation coefficient. To avoid this while having a measurement of the variability, we applied a bootstrap strategy where we reduced the variation caused by the generation number and by differences in colony identity (Fig. S4B,C, see Supplementary Materials and Methods).

Our results show a strong and statistically significant correlation of the cell cycle variables between sister cells, both in the naive ground state and in the transition to formative pluripotency (Fig. 3B). Interestingly, the coefficient of correlation was significantly higher for the length of the G1 phase compared to the length of the S/G2/M phases. This strongly suggests the existence of inherited determinants of the cell cycle from the parental cell, which are similarly shared by sisters after the symmetrical cell division. Furthermore, while G1-L could be greatly determined by these inherited factors, the requirement for biological processes such as RNA transcription and protein synthesis in the transition to the S/G2/M phases could explain why this correlation is significantly decreased in these phases.

Next, we wondered whether there was also a strong correlation between parental and daughter cells. We computed the Spearman coefficient for each variable between each parental cell and one of its daughters chosen at random. We observed a small but significant correlation in the three variables for differentiating cells, and also in the case of G1-L and CC-L for undifferentiated cells. Overall, the low correlation suggests that the length of the cell cycle phases of daughter cells, although probably governed by some yet unknown factors inherited from the mother, is not directly linked to the length of the parental cell cycle phases.

Finally, we analyzed the correlation between cousin cells and found a significant but low correlation in the G1-L, in both conditions. This coefficient was higher than the expected ${\rho }_{c-c}={{\rho }^{2}}_{m-d}\times {\rho }_{s-s}$ from simple inheritance rules, as has been recently reported in another cell system^24^. This could indicate that although mother and daughter cells are not highly correlated, there could still be inherited deterministic factors that could influence cell cycle variables. To analyze this behavior in a complementary way, we studied whether the cell cycle variables within cell lineages were governed by stochastic or deterministic factors. As proposed by Sandler *et al*., we applied the Grassberger-Procaccia algorithm to G1-L and SG2M-L^26^. Interestingly, this analysis indicated the existence of deterministic factors in the cell cycle variables that were inherited from mother and even grandmother cells (Fig. S5, see Supplementary Materials and Methods). This is consistent with both the high correlation between sister cells for G1-L and the obtained inequality ${\rho }_{c-c} > {\rho }_{m-d}^{2}\times {\rho }_{s-s}$. The low mother-daughter correlation is not against the Grassberger-Procaccia results, which is more general because it takes one more generation (grandmother cell) into account. If there is a deterministic factor inherited from the grandmother cell, as suggested by our results, then the mother inheritance could not be enough to completely determine a phase length, yielding a low mother-daughter correlation.

### Transition from naive to formative pluripotency results in changes in cell movement and morphology

**Figure 4:**
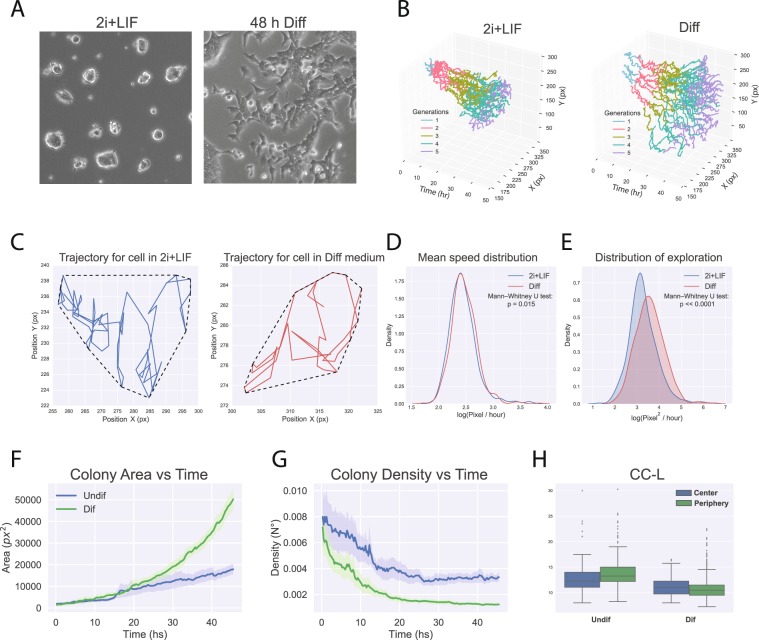
Morphological reorganization of mESCs during the transition to formative pluripotency. (**A**) Morphology of cells cultured in 2i + LIF or after 48 h of culture in Diff medium. (**B**) Individual cell trajectories over time for cells within representative colonies maintained in 2i + LIF or in Diff medium. Cell generation numbers are indicated in colors. (**C**) Blue and red lines show spatial trajectories for representative cells cultured in ground state conditions or in Diff medium. Dashed lines surrounding the trajectories depict their convex hull. (**D**) Distribution of mean cell speeds for cells grown in 2i + LIF or in Diff medium, calculated for each cell as the total trajectory divided by the CC-L. N = 516 and 623 cells for naive ground state and differentiation, respectively. Data was transformed to log scale for visualization and analyzed for statistical differences with a Mann Whitney U test. (**E**) Distribution of cell exploration for cells grown in 2i + LIF or in Diff medium, calculated for each cell as the convex hull area divided by the CC-L. Data was transformed to log scale for visualization and analyzed for statistical differences with a Mann Whitney U test. (**F**) Colony area vs Time for colonies maintained in 2i + LIF or in Diff medium. N = 11 and 12 colonies for 2i + LIF and Diff medium, respectively. Shaded areas show the 95% confidence interval. (**G**) Colony density vs Time for colonies maintained in 2i + LIF or in Diff medium. N = 11 and 12 colonies for 2i + LIF and Diff medium, respectively. Shaded areas show the 95% confidence interval. (**H**) CC-L distribution for center and peripheral cells in 2i + LIF or in differentiating conditions.

During the implantation of the mouse embryo, the cells of the pre-implantation epiblast rapidly transform into a cup shaped epithelium, the post-implantation epiblast^27^. We thus decided to analyze if the *in vitro* transition from naive ground state pluripotency to formative pluripotency is also accompanied by an important morphological reconfiguration. mESCs in the ground state grow as tightly packed colonies with a dome shape. We observed that during the transition to formative pluripotency colonies rapidly lost this morphology (Fig. 4A). To analyze this behavior in more detail, we took advantage of the positional data gathered on each cell in both culture conditions. This allowed to faithfully reconstruct the organization of colonies over time (Fig. 4B). We wondered if the difference in colony organization could be accounted by an increased mobility of cells when induced to differentiate. To analyze this, we first evaluated if there were any differences in the velocity of cell movement. We calculated the trajectories of cells in both conditions (Fig. 4C). Then, by knowing the CC-L of each cell, we determined the mean speed distribution of cells in each condition Interestingly, this analysis showed a statistically significant difference between the mean velocity of cells (p < 0.05), with differentiating cells moving at a slight higher rate than cells in 2i + LIF (Fig. 4D). We next wondered if the different organization of colonies could be associated to an increased movement of cells within their surrounding area. To quantify this, we calculated the convex hull of the trajectory for each cell as a measure of how much area they explored and normalized it by the CC-L to avoid a bias induced by differences in cycling time (See Fig. 4C, dashed lines). This analysis showed that differentiating cells indeed displayed a statistically significant increase in the area of exploration, which, interestingly, was more pronounced in cells of later generations (Figs 4E, S6A). Furthermore, we observed a reorganization of the actin cytoskeleton of differentiating cells that suggested that these cells are more tightly attached to the plate substrate (Fig. S6B). In agreement with this, cells in Diff medium displayed a larger apparent nuclear area that was already evident in the 2nd cell generation, consistent with a shift towards stronger adherence and epithelial phenotype (Fig. S6C). As a result, differentiating colonies progressively displayed greater areas and lower cell densities over time (Fig. 4F,G).

### Cell cycle length is similar between cells of the center and periphery of the colony

We finally evaluated the existence of differences in the cycle variables of cells with respect to their position within the colonies. Indeed, recent publications have shown that mouse and human ESCs display different developmental capacities that are linked to their relative location within the colony^28–30^. Since the cell cycle has been linked to stem cell properties, we decided to analyze if cells displayed different cell cycle properties with respect to their position. To do so, we evaluated the CC-L distribution of cells in the periphery and in the center of the colony. We considered cells as “peripheral” when, at a given moment of their life, they were in contact with the convex hull of the colony. This analysis, however, showed no significant differences in the CC-L of cells regarding their position (Fig. 4H). Interestingly, these results are in line with the high culture homogeneity in the naïve ground state and during transition to formative pluripotency.

## Discussion

Mammalian pluripotent stem cells provide an extraordinary experimental system to study embryonic development and are an important promise in areas such as regenerative medicine, drug discovery and tissue engineering. During early embryonic development, cells of the peri-implantation epiblast have the capacity to proliferate at unusually rapid rates^31^. This phenomenon is recapitulated in mESCs cultured in FBS/LIF medium, which possess a reported cell cycle length of 11–14 h, and a length of the G1 phase of approximately 2 hours^21,25,32^. The cell cycle structure and molecular properties of mESCs in serum-based media have been extensively studied, which has allowed deciphering key regulatory properties that distinguish them from other somatic cell types. However, after the advent of new culture systems that allow maintenance and derivation of mESCs in the naive ground state of pluripotency, an in depth characterization of the cell cycle of mESCs in this state and during its dissolution has been lacking^6^. A number of reports have indirectly measured the cell cycle length of mESCs in the ground state, although with important discrepancies. It has been suggested that mESCs cultured in media containing FBS/LIF or 2i + LIF grow at similar rates^33^, while another work reported a significantly higher cell cycle length of approximately 25 h in the ground state compared to the 11 h in serum based media^34^. Although a recent report has showed that addition of 2i to mESCs cultured in FBS/LIF (FBS/LIF/2i) causes a slight but significant lengthening of the cell cycle^25^, this culture condition does not represent the ground state of pluripotency. In this work, by performing live imaging experiments of FUCCI mESCs in the naive ground state, we directly measured the cell cycle length, the length of the G1 phase and the length of the S/G2/M phases of hundreds of individual cells. Contrary to other methods that rely on measuring indirect variables on the overall population such as propidium iodide staining or nucleoside analog incorporation^35^, our analysis allowed to capture the complete distribution of these variables at the single cell level, providing a more detailed characterization of the cell cycle dynamics. Our results suggest that mESCs grown in the naive ground state display a similar proliferation rate than the previously reported for cells cultured in FBS/LIF.

It has been widely shown that when mESCs exit from the pluripotent state, the cell cycle structure is reorganized, and both G1 and the total cycle are lengthened. This has also been observed during the early onset of differentiation, both in the transition to primed EpiSCs and in differentiation protocols that rely on LIF withdrawal^3,21^. Here, however, we demonstrate that transition to formative pluripotency is rapidly associated to an overall shortening of both G1 and S/G2/M, causing a shorter cell cycle length and thus an increased proliferation rate. These results are compatible with a previous report where mESCs were differentiated into early primitive ectoderm-like cells (mEPLs) using an undefined FBS containing conditioned medium of HepG2 cells^32^. The authors observed that when mESCs cultured in FBS/LIF were differentiated to mEPLs, cells had an increased proliferation rate, as analyzed by propidium iodide staining. However, this experimental system has not been extensively validated and the use of an undefined conditioned medium to induce differentiation presents major disadvantages compared to current protocols. In spite of this, the fact that two analogous differentiation protocols that induce an early post-implantation identity present higher proliferation rates challenges the idea that the exit from naive pluripotency is directly coupled to a lengthening of the cell cycle, as previously suggested^3^. Instead, we propose that transition to formative pluripotency is associated to an increased proliferation rate that is reduced as cells reach primed pluripotency and lineage commitment. This view is compatible with the higher proliferation observed in post-implantation epiblast cells of the mouse blastocyst, which is only reduced when epiblast cells reach the gastrulation stage^31,36^.

A key advantage of time lapse imaging compared to other methods is that it allows to obtain single cell measurements on complete cell lineages. Using this methodology, it was recently observed in lymphocytes and in mESCs cultured in FBS/LIF that sister cells present highly correlated length of the cell cycle, which suggests the parental inheritance of cell cycle determining factors^24,25^. With a similar analysis, we observed that this is also true for mESCs cultured in the naive ground state and during transition to formative pluripotency. Moreover, the correlation of G1 length between sisters was significantly higher than the correlation of S/G2/M. This strongly supports the existence of inherited determinants of the cell cycle from the parental cell, which are similarly shared by sisters after the symmetrical cell division. Furthermore, while G1-L could be greatly determined by these yet unknown inherited factors, the requirement for biological processes such as RNA transcription and protein synthesis in the transition to the S/G2/M phases could explain why this correlation is significantly decreased in these phases. Lineage correlation analysis also suggested a mild level of correlation between mother-daughter and cousin cells, which indicates that cell cycle determining factors are partially inherited for at least a few generations.

Finally, we decided to characterize the morphological changes that occur during the dissolution of ground state pluripotency. Many reports had previously observed a major transformation from the compact colonies of mESCs to more dispersed cell structures^15,37^, but a detailed characterization of these events had not been performed. By taking advantage of the gathered data on cell position, we show that induction of differentiation rapidly changes the behavior of individual cells by increasing their overall mobility. Interestingly, we also observed a transformation in the nuclear morphology which correlated with an increased adherence to the culture surface. This morphological reconfiguration is supported by recent RNA-seq experiments, that show increased expression of genes involved in cell adhesion during the transition to formative pluripotency^14^. Furthermore, this transformation is in line with events that take place during embryonic implantation, where the amorphous epiblast undergoes a morphogenic transformation into a columnar epithelium^36^.

The discovery of the different pluripotent cell types that can be generated *in vitro*, together with recent advances in cell culture techniques have made a profound impact in the field of pluripotent stem cell biology. Efficient differentiation of mESCs into another pluripotent cell type that resembles the next developmental stage provides a remarkable *in vitro* model to study the genetic and epigenetic mechanisms that regulate cell identity transformation. We believe that the results presented in this work contribute to a greater understanding of the cell cycle events that take place in the transition from ground state to formative pluripotency.

## Materials and Methods

### Cell culture and differentiation

W4 Fucci-H2B mESCs generation was previously described^18^. Ainv15 cells were purchased from ATCC, and 46 C mESCs were a kind gift from Dr. Austin Smith. Cells were cultured using the chemically defined medium N2B27 with 1000 U/ml LIF (Millipore), 1 µM PD0325901 (Tocris) and 3 µM CHIR99021 (Tocris), hereafter called ‘2i + LIF medium’. N2B27 formulation is described elsewhere^38^. Cells were maintained on 0,1% gelatin coated dishes, passaged every three days using TrypLE (Gibco) and cultured at 37 °C in a 5% CO2 (v/v) incubator. Cells were cultured without antibiotics and routinely assessed for mycoplasma contamination by PCR. To induce differentiation from the naive ground state to formative pluripotency, medium was replaced for N2B27 prepared with regular B27 supplement (GIBCO) and in the absence of LIF and 2i (‘Diff medium’). Cells were grown for 24–48 h.

### Live imaging

For time lapse experiments, cells were plated in µ-Dish 35 mm dishes (IBIDI) previously coated for 1 h with poly-D-Lysine and then for 2 h with 521-laminin (BioLamina) at 37 °C. Cells were seeded at a density of 1 × 10^4^ cells/cm^2^ and grown for 48 h in 2i + LIF medium. Before starting the live imaging experiments, cells were washed two times with PBS 1X and switched to Diff medium or maintained in 2i + LIF medium. Time lapse experiments were performed on the CellVoyager spinning disc confocal microscope (Yokagawa/Olympus) at 37 °C in 5% CO_2_ (v/v). Individual cell colonies were selected and imaged for 45 h at 20X at 4 different positions (tiles) that were later automatically stitched to form a larger image. Images were acquired every 15 minutes with 10 Z-slices on different channels to record H2B-mCerulean, hGeminin-mVenus and hCdt1-mCherry fluorescence. Laser intensity and exposure time were minimized to avoid phototoxicity. Data for ground state mESCs and differentiating cells was gathered from 4 and 2 independent time lapse experiments, respectively.

### Nuclear segmentation, cell tracking and data extraction

Maximum intensity projections were generated from the z-stacks of each channel and time point. Background was subtracted from each channel and image stacks were fed into the *LinageTracker* plugin of Fiji/ImageJ^39^. Nuclear segmentation was carried out using the H2B-mCerulean fluorescence by a threshold-based binary mask and then applying the watershed method. Incorrect nuclear segmentations were manually corrected. Nuclear and cell division tracking were automatically performed and erroneously assigned tracks were manually corrected. *LineageTracker* results consist of multiple text files containing information of individual cells overtime, such as area, fluorescence, position and also the identity of daughter cells. To interpret this complex data structure, we generated a custom Python script that automatically reconstructs cell lineages and determines the cell cycle variables CC-L, G1-L, and SG2M-L for each complete cell. This script provides a graphical interface for the visualization, annotation and data correction of individual lineages and cells. It also allows to manually annotate different features (e.g. apoptosis, polyploidy) and to correct G1-L and SG2M-L in the case that automatic detection of fluorescent peaks on individual cells fails. After manually curating and validating the data, the script generates a convenient data base that is ready for downstream analysis with R, Python or other programming languages. The Python script for processing and validating *LineageTracker* data is available upon request. Data extracted from the time lapse imaging is available in the Supplementary File.

### Data analysis and statistics

All data analysis was performed in Python. Statistical differences were considered significant when p-values were lower than 0.05. Comparison of data distributions between 2i + LIF and Diff were analyzed with the non-parametric Mann Whitney U test. Differences in the length of the cell cycle phases between cells of different generations were analyzed with a log rank test based on the Kaplan Meier results. Correlations within lineages (sisters, mother-daughter and cousin cells) were calculated using a bootstrap strategy. For detailed information about the analyses, see the Extended Supplementary Materials and Methods. Comparison of the proportion of S-phase cells after EdU incorporation was between experimental conditions was performed by randomized block design ANOVA.

### Immunofluorescence

Actin filaments in mESCs and differentiating cells were stained using Texas Red™-X Phalloidin (Thermo Fisher Scientific), according to manufacturer’s instructions.

### EdU incorporation and propidium iodide staining

We assessed the proportion of cells in the S-phase by incorporation of 5-ethynyl-2′-deoxyuridine (EdU) using the Click-iT™ EdU Alexa Fluor™ 647 Flow Cytometry Assay Kit (Thermo Fisher Scientific), following manufacturer’s instructions. Cells were pulsed with 10 µM EdU for 30 minutes and, after the click-it reaction, cells were stained with 25 µg/ml propidium iodide (Sigma).

## Supplementary information

Video S1

Supplementary Information

Supplementary File
