# Supplementary material for: Cell cycle dynamics of mouse embryonic stem cells in the ground state and during transition to formative pluripotency
Source: Sci Rep. 2019 May 29;9:8051. doi: 10.1038/s41598-019-44537-0 (PMC6541595; doi:10.1038/s41598-019-44537-0)
Supplement: Supplementary file 2 — Supplementary Information [file 41598_2019_44537_MOESM2_ESM.pdf]

Supplementary Information

Cell cycle dynamics of mouse embryonic stem cells in the ground state and during transition to formative pluripotency

Ariel Waisman[1,2], Federico Sevlever[3], Martín Elías Costa[4], María Soledad Cosentino[1], Santiago G. Miriuka[2], Alejandra C. Ventura[3], Alejandra S. Guberman[1,5,*]

Supplementary Figures

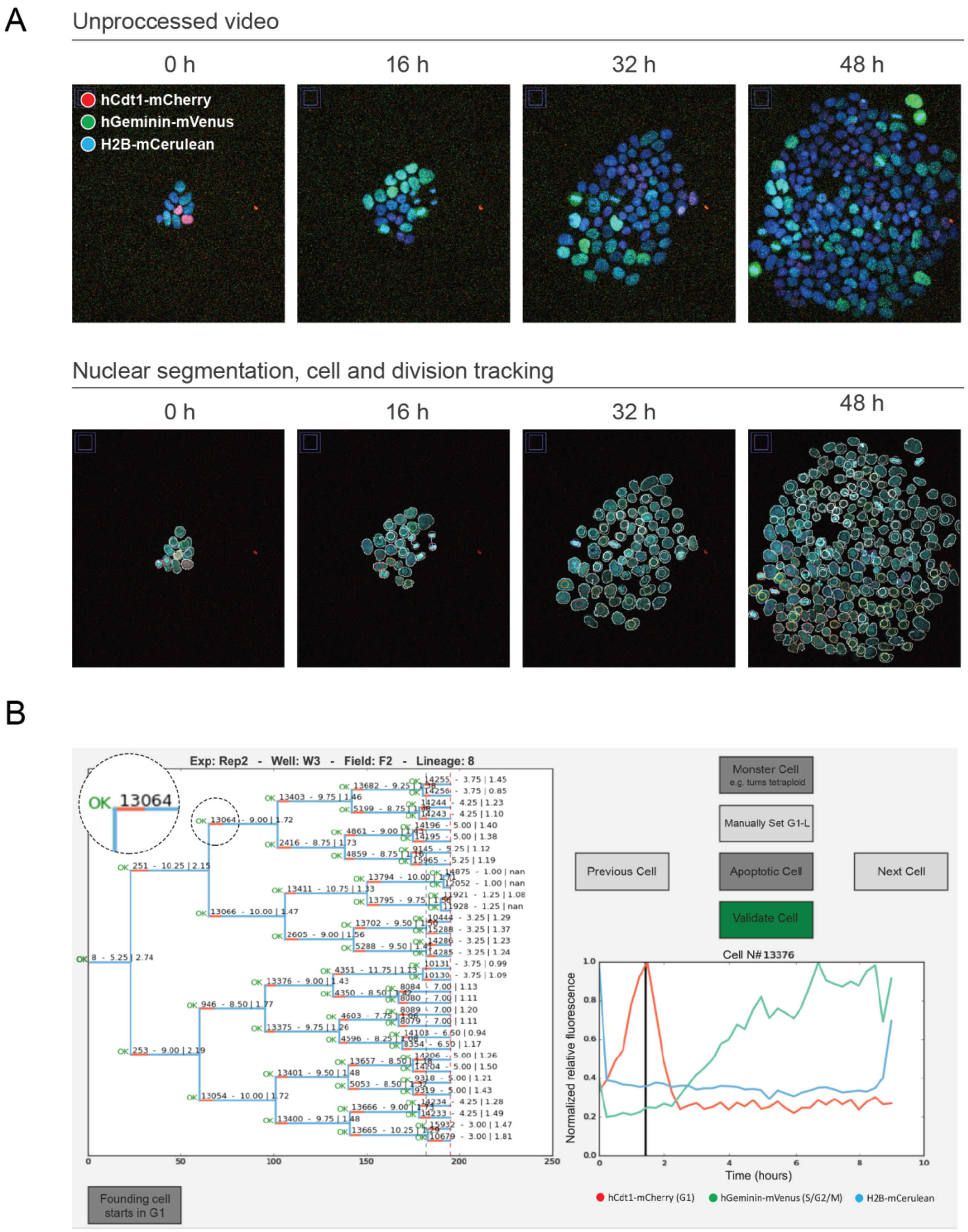

Fig. S1. Nuclear segmentation, cell and division tracking, and graphical interface for data validation. (A) *Upper panel*, unprocessed images from a time lapse experiment of a colony maintained in naive ground state conditions. Images are merged compositions for the three channels corresponding to hCd1-mCherry, hGeminin-mVenus and H2B-mCerulean. The *lower panel* displays the same colony after manually corrected automatic nuclear segmentation, cell tracking,

and division tracking using the *LineageTracker* plugin of imageJ. (B) Graphical interface developed for the visualization, annotation and data correction of individual lineages and its composing cells. The script allows to interpret the data produced by *LineageTracker* and automatically generates the lineage dendrogram, while also automatically determining the cell cycle variables CC-L, G1-L, and SG2M-L for each cell that completed the cell cycle during the time lapse imaging. After manually validating each cell and annotating different features (e.g. apoptosis, polyploidy), the script generates a convenient database that allows further analysis.

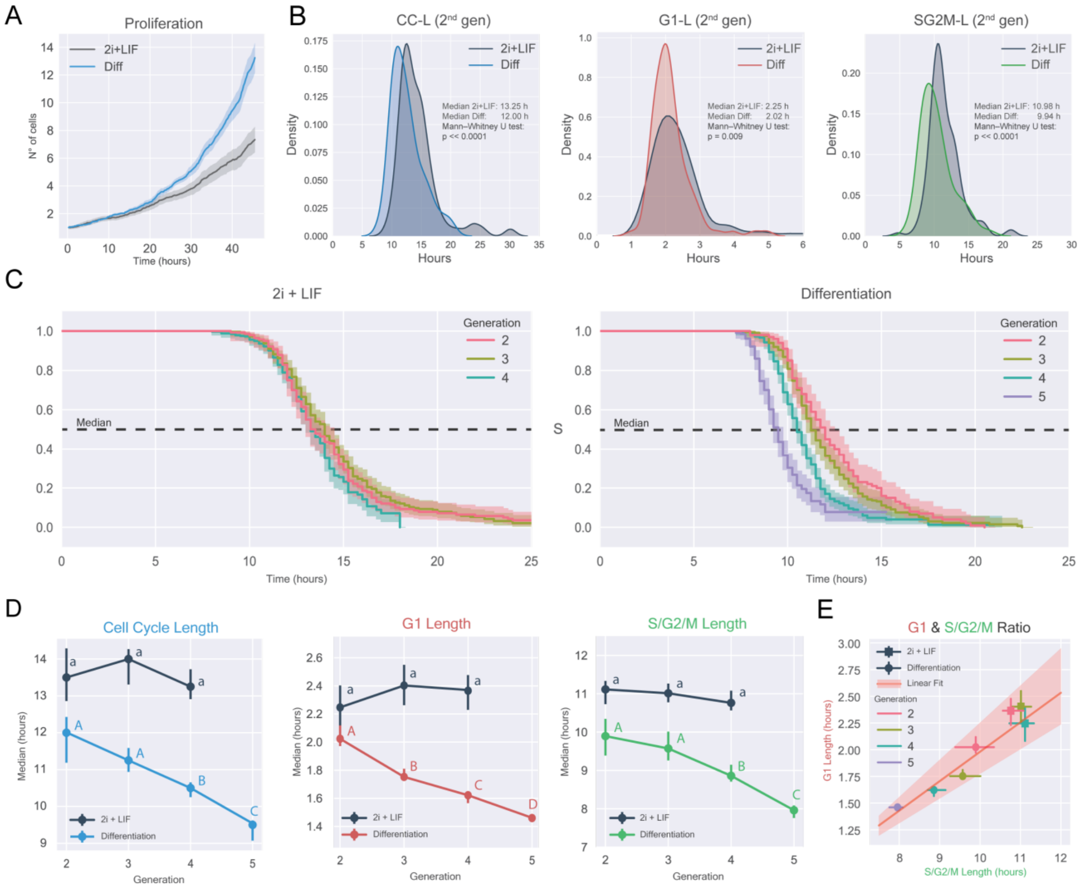

Fig. S2. Proliferation rate of mESCs in ground state conditions and during differentiation. (A) Mean number of cells as a function of time among lineages. 72 and 50 lineages were analyzed for 2i+LIF and differentiation, respectively. Light blue and light gray areas represent the 95% confidence interval. (B) Density distribution for the variables CC-L, G1-L and SG2M-L for the cells in the second generation after the beginning of the live imaging, comparing cells maintained in the naive ground state and during the transition to formative pluripotency. N = 139 and 100 cells for naive ground state and differentiation, respectively. Statistical differences were assessed with a Mann Whitney U test. (C) Kaplan Meier survival functions of time, grouped by generations for 2i+LIF (left panel) and differentiation (right panel). The CC-L distribution median is the time where the survival probability cross 50%. Shaded areas represent the 95% confidence interval for each curve. (D) Median values for CC-L (left panel), G1-L (center panel) and S/G2/M-L (right panel) deduced from the Kaplan Meier survival analysis, as a function of generation. Black curves represent 2i+LIF condition and blue, red and green represent differentiation condition for CC-L, G1-L and S/G2/M-L, respectively. Statistical differences were assessed with a log rank test. Different letters indicate significant differences between groups ($p < 0.05$). (E) G1-L vs. S/G2/M-L median values for each generation and both

conditions (2i+LIF, square dots; differentiation, circle dots). Error bars represent the 95% confidence interval. Red line and light red represent a linear fit with the 95% confidence interval of the ratio.

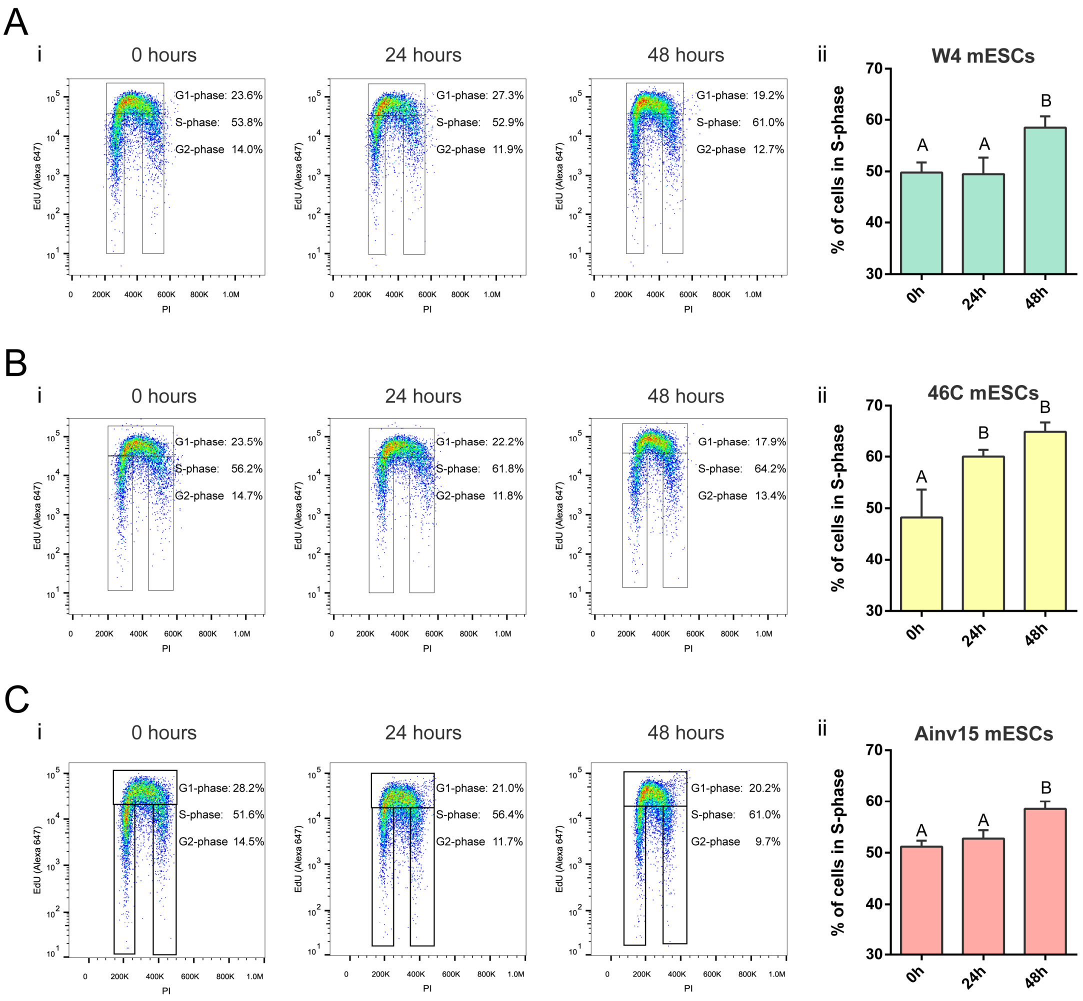

Fig. S3. Evaluation of proliferation rate by EdU incorporation and propidium iodide staining in different cell lines. (A) i. Representative examples of flow cytometric analysis of EdU incorporation and PI staining of W4 mESCs at 0 h (2i+LIF), 24 and 48 h after the differentiation stimulus. Edu+/- gates were determined with controls in which cells were not pulsed with EdU. ii. The chart shows the percentage of cells in the S-phase for the three conditions under analysis and for three independent biological replicates. Results are shown as mean ± SEM. Different letters indicate significant differences between groups ($p < 0.05$) by a randomized block design ANOVA. (B) and (C), similar to (A) but for 46C mESCs and Ainv15 mESCs, respectively.

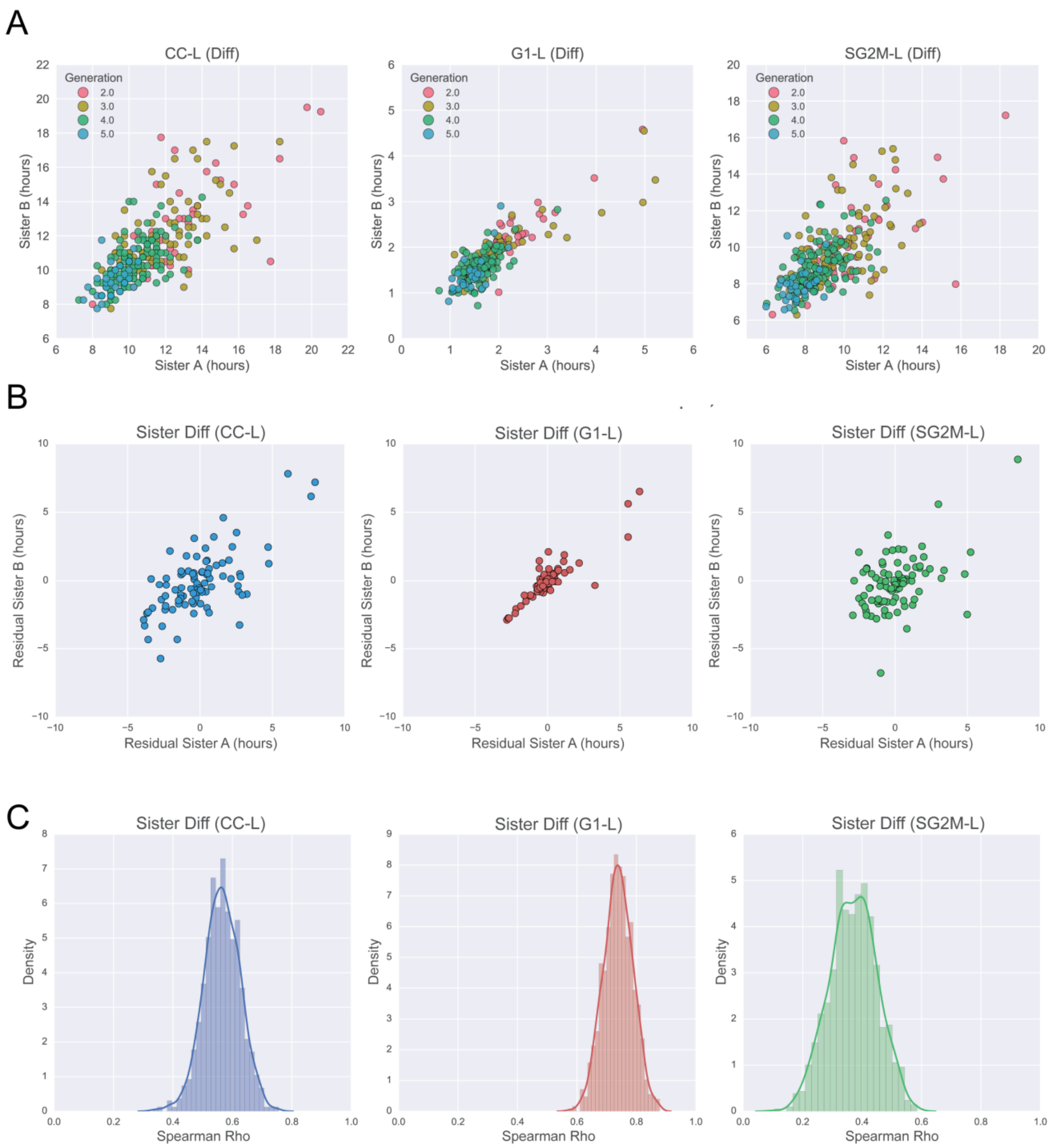

Fig. S4. (A) Correlation plots for the CC-L, G1-L and SG2M-L variables for pairs of sister cells cultured in differentiating conditions. Generation numbers are indicated in colors. (B) Representative iteration of the bootstrap analysis showing a correlation plot for the residual CC-L, G1-L and SG2M-L of sister cells in differentiating condition with respect to the colony generational mean and with a balanced distribution of cells in the different generations (see Materials and Methods). (C) Distribution of Spearman coefficients for the correlation of the CC-L, G1-L and SG2M-L of sister cells in differentiating conditions after 1000 bootstrap iterations. Median Spearman coefficient and the 95% confidence interval were obtained from this analysis.

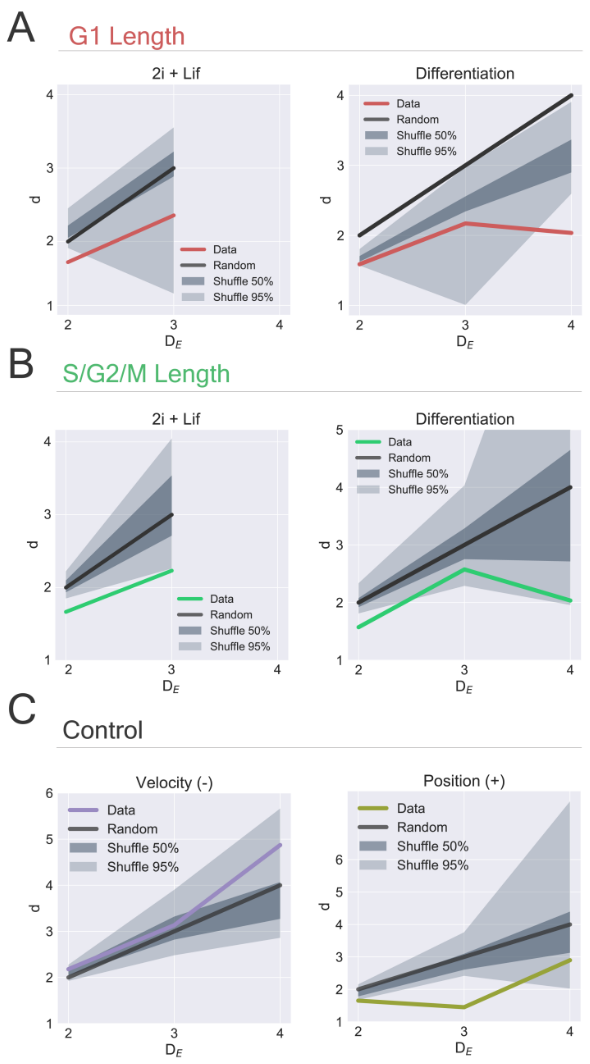

Fig. S5. Grassberger-Procaccia results. Black lines represent the random control while light and dark gray represent the 50% and 95% confidence interval of shuffled data for all panels. All data values (colored lines) are below random controls and between 2 and 3, supporting a deterministic lineage inherited factor (see Supplementary Materials and Methods) (A) G1-L data values (red lines) for 2i+LIF (left panel) and differentiation (right panel). (B) SG2M-L data values (green lines) for 2i+LIF (left panel) and differentiation (right panel). (C) Negative and Positive controls using cell's Velocity (left panel) and Position (right panel), respectively. Both controls behaved as expected.

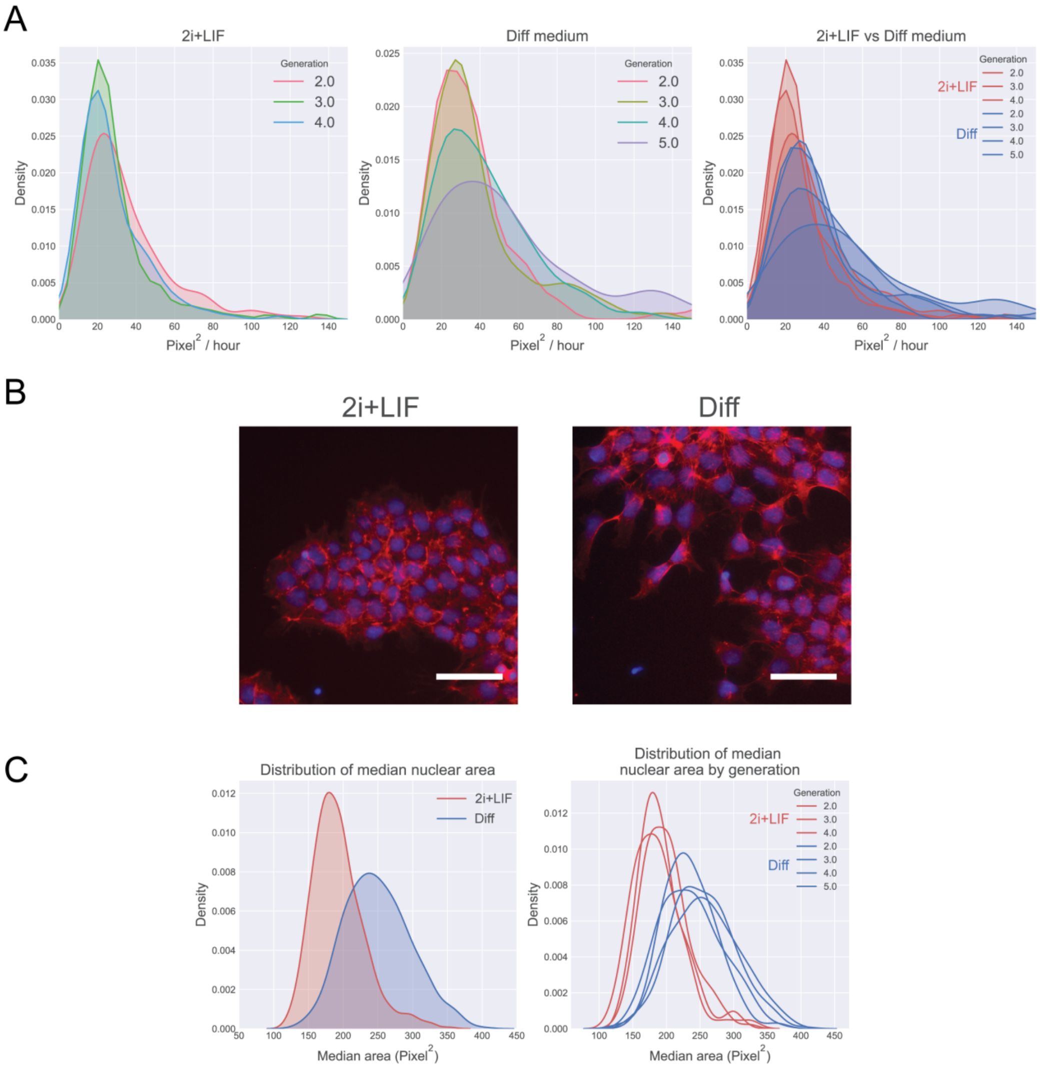

Fig. S6. Morphological changes during the transition to formative pluripotency. (A) Distribution of cell exploration for mESCs in 2i+LIF or differentiating cells (Diff) according to the cell generation number, calculated for each cell as the convex hull area divided by the CC-L. (B) Reorganization of the actin cytoskeleton during differentiation. Texas-Red Phalloidin (red) and DAPI (blue). Scale bar, 50 µm. (C) Distribution of apparent nuclear area in cells cultured in 2i+LIF or in differentiating conditions for all cells (left) or for cells grouped according to their generation number (right). N = 516 and 623 cells for ground state and differentiation conditions, respectively.

Video S1. Representative time-lapse of differentiating cells. The video shows a mESCs colony growing for 45 hours in Diff medium (i) without processing, (ii) with segmentation of cell nuclei, and (iii) with segmentation, nuclear tracking and cell division tracking.

Extended Supplementary Materials and Methods

Data analysis

All data analysis was performed in Python. For most of the analyses, only cells that completed its cycle ("complete cells") were considered, i.e., cells with a recorded time of birth and time of division.

Estimation of unbiased median length of the variables CC-L, G1-L and SG2M-L

Estimation of unbiased median length of the variables CC-L, G1-L and SG2M-L was performed by calculating the survival function using the Kaplan Meier method (Goel et al 2010). This method considers the incomplete cells of the video by estimating the cumulative distribution of the variable. For example, for CC-L, it is impossible to calculate the distribution value of a given time because of the incomplete cells which have not a CC-L observed. If these cells are discarded, then there will be a bias in the distribution and its mean value. However, the cumulative distribution can be estimated by counting the number of divisions over the total number of cells (even the incomplete ones). This provides an approximate cumulative distribution from where the median can be obtained despite the incomplete cells (see Fig. S2C).

Estimation of spearman correlation coefficients using a Bootstrap strategy.

To calculate the Spearman correlation coefficients reducing the bias induced by generation number and between cells of different colonies, we applied a bootstrap strategy[1]. Briefly, we calculated the residuals of the cell cycle variables for each cell with respect to the generational mean within each colony. To avoid oversampling cells of later generations, we randomly selected an even number of pairs of sister cells, mother-daughter or cousin cells among the different generations for the different colonies analyzed, and calculated a preliminary Spearman coefficient for that iteration. We repeated the random sampling with replacement 1000 times and calculated the median Spearman coefficient together with a 95% confidence interval (see Fig. S3 B and C). This analysis allows to obtain the p-values for a Spearman rho greater than 0 by calculating the number of cases in which the Spearman rho of a given iteration was ≤ 0 and dividing it by the total number of iterations.

Grasberger Procaccia algorithm

The Grassberger-Procaccia algorithm was first applied to study lineage inheritance by Sandler et al[2], where is explained in detail. The general aim of the analysis is to detect if there is a

deterministic relationship between any groups of different variables. For any kind of data from 2 independent and uncorrelated variables, when plotting one against the other, data will be randomly distributed in the 2-dimensional plane, occupying all the space with some nearly constant density. Conversely, if there exist a relationship and one variable determines the other, data will shape a curve in the plane. To make the distinction between a curve and a uniform density one can imagine an expanding circle centered on one data point and just count the number of points inside this circle as a function of the radio of the ball *r*. If the variables are independent, this number will increase proportionally to the area of the circle, to *r^2*. But in the case of a deterministic relation, this number will be proportional to the length of the curve locked inside the ball, this means, with *r^1*. The final step of the algorithm is to fit the number of points inside the circle, as a function of the circle radio, to extract the radius exponent and see if it is equal (independent) or lower than 2 (deterministic).

This same idea can be extended to 3 dimensions or more. For example, in 3 dimensions for 3 independent variables, data points will be distributed in all the space and points inside a sphere will increase as *r^3*. However, there is another case where two variables, instead of one, determine the other. In this case, data points will shape a plane in a 3-dimensional space, making the number of points inside the ball increase proportionally as the plane locked area, *r^2*. The final step now is the same as before, where if the radius exponent is equal to 3, the 3 variables are independent, but if it is lower there is a deterministic factor relating them.

In the case of more dimensions (more variables), the conclusion is made just by looking if the radius exponent, *d*, is equal or lower than the embedding dimension (the number of variables), *DE*. To study lineage inheritance, the algorithm is applied to 2, 3 and 4 variables ascending in lineage progenitor generations. For example, we take data of G1-L of one cell and G1-L of its mother cell as the two variables to study if there is an inheritance factor that makes G1-L of the mother cell to determine G1-L of daughter cell. Another possibility is that the determination is made by both mother and grandmother cells, so we extended the analysis with the G1-L of the grandmother cell and applied the algorithm again. This process is extended back in the lineage until the results show that either *d* reaches a fixed value independent of *r*, or it continues increasing equally to *DE*. In this second case, the conclusion is that G1-L of daughter cells is independent of cell lineage or that we must look even further back. On the other hand, if *d* reaches a fixed value, this value is near to the number of generations that determines the daughter cell's G1-L. For example, if *d=2*, then it is determined by the mother and grandmother cells.

The Grassberger-Procaccia plots (Fig. S4) shows d for the different values of *DE*, where it can be seen that *d* is always lower than *DE*. However, it is important to quantify how much the difference

between *d* and *D* has to be to conclude that there is a determination. For this purpose, we developed a statistical analysis which consists on randomly mixing all lineages and then applying the algorithm to this shuffled data and repeat the process several times. This procedure provides a distribution of *d* values whose mean value, compared to *DE*, indicates if there is no external determination. This distribution corresponds to free lineage relations data and represents our null hypothesis, which we can compare to the real data. We decided to take the confidence intervals of these shuffled data distributions instead of the standard deviation because of the asymmetry of the distributions obtained.

Finally, we performed a positive and a negative control of the Grassberger Procaccia algorithm (Fig S4 C). We applied the algorithm to the data of each cell´s first position in the video and also to the mean velocity of cells. As cells "born" from its mother in a close position, this variable works as one determined from mother and the analysis confirms it (positive control). On the other hand, we expected mean velocity to be a purely stochastic variable, which was also confirmed (negative control).

Supplementary References

1. Efron, B. & Tibshirani, R. J. An Introduction to the Bootstrap. *Refrig. Air Cond.* **57,** 436 (1993).
2. Sandler, O. *et al.* Lineage correlations of single cell division time as a probe of cell-cycle dynamics. *Nature* **519,** 468–471 (2015).
